# Shallow foundation design: a comparative study of partial safety factors and full probabilistic methods

**DOI:** 10.1038/s41598-024-63003-0

**Published:** 2024-06-13

**Authors:** Miroslav Vořechovský, Lumír Miča, Jiří Boštík

**Affiliations:** 1 Faculty of Civil Engineering, Institute of Structural Mechanics, Brno University of Technology, Veveří 331/95, 602 00 Brno, Czech Republic; 2 Faculty of Civil Engineering, Institute of Geotechnics, Brno University of Technology, Veveří 331/95, 602 00 Brno, Czech Republic

**Keywords:** Civil engineering, Statistics

## Abstract

In the past two decades, Europe has witnessed a significant transition in the design codes used for assessing foundation structures, with the widespread adoption of the Eurocodes (EC). This shift remains a pertinent topic within the engineering community, particularly concerning the transition from traditional design methodologies to those prescribed by the Eurocodes, as well as the potential for fully probabilistic design. While the Eurocodes’ methodology is described as probabilistic, it is crucial to recognize that the achievement of the target reliability level is predominantly facilitated through a system of partial safety factors. These factors are integrated into the calculation algorithm as fixed values, rendering the process essentially deterministic. To refine these calculations for more accurate reliability estimates—expressed in terms of failure probability—a genuinely probabilistic framework is required, termed as fully probabilistic computation. This paper aims to elucidate the fully probabilistic calculation approach for the broader professional community, using the geotechnical application of shallow foundations as an illustrative example. We present a comparative analysis of this advanced approach with the standard foundation design according to EC7 and ČSN 731001, the latter being a precursor in Europe for implementing the partial safety factor method. The discussion extends to a practical demonstration of full probabilistic design juxtaposed against the conventional partial safety factor method, using a shallow foundation case study. Furthermore, the paper delves into the impact of the tail behavior of uncertain or spatially varying soil parameters on the theoretical probability of failure, underscoring its significance in foundation design.

## Introduction

In contemporary engineering practice, it is universally acknowledged that effective modeling and analysis of uncertainty, as well as the assessment of its impact on safety and design, fundamentally rely on core probabilistic concepts. This paradigm shift, recognized in seminal works such as^1^, posits that under conditions marked by uncertainty, the assurance of safety and serviceability in structures is best expressed in probabilistic terms. Specifically, it is through the lens of the probability of survival, or inversely, the probability of failure, that these aspects are most accurately evaluated and managed.

Reliability-Based Design (RBD) is a prevalent practice in the structural engineering community globally. However, its adoption in geotechnical engineering varies internationally. RBD is adopted in North America, Japan, and the Netherlands, the partial safety factor method is adopted in European Union, the United Kingdom, while the global factor of safety via allowable stress design is more common in other parts of the world^2^.

The Terzaghi Lecture, a prestigious series with a rich tradition spanning over 60 years, has featured several presentations that have significantly contributed to the discourse on risk and reliability in geotechnical engineering. Notably, Casagrande^3^ delivered the first lecture in this series on the topic in 1965, setting a foundational precedent. This was followed by an influential presentation by Whitman^4^ in 1984, among others, culminating in the most recent lecture by Baecher^5^. These lectures, part of a select few that have addressed risk and reliability since the series’ inception, highlight the evolving understanding and importance of these concepts in the field. The substantial body of literature on geotechnical risk and reliability that has emerged since the 1960s is a testament to this growth^6–12^, with Phoon^2^ providing a recent comprehensive review, including extensive references. This chronology underscores the ongoing significance and progressive development of risk and reliability considerations in geotechnical engineering.

The Factor of Safety (FoS) method historically represented a foundational approach to assess the reliability of engineered structures. Predominating traditional engineering design, this method’s appeal lay in its simplicity and direct applicability. It involved applying a single safety factor to account for various uncertainties, providing a straightforward measure of structural reliability. However, the FoS method, despite its widespread use, lacked the detailed nuance and specificity found in subsequent methodologies. With the advancement in engineering practices, there was a shift towards the partial safety factors method and eventually, the adoption of full probabilistic design. These later methodologies offer a more sophisticated understanding of uncertainties in engineering design. They consider the probabilistic nature of various factors like material properties, environmental conditions, and loading scenarios. This evolution in design philosophy reflects an increased emphasis on precision and risk management in engineering. It acknowledges that different aspects of a structure may have varying levels of uncertainty and that a one-size-fits-all approach like FoS may not adequately address these nuances. The move towards more probabilistic methods represents a significant advancement in the field, aiming to enhance the safety, efficiency, and reliability of structural designs.

The Eurocodes, a comprehensive suite of standardized structural design codes, are pivotal in building and civil engineering works across Europe. These codes aim to ensure a high level of reliability in structural designs, thereby minimizing failure risks and protecting human life, economic investments, and the environment. Comprising 10 independent documents, the Eurocodes address various structural types (except for the first two documents).

Specifically, geotechnical structures are covered under EN 1997, known as Eurocode 7: Geotechnical Design. Eurocode 7 (EC7) is divided into two main parts: Part One, which outlines General Rules^13^, and Part Two, which focuses on Ground Investigation and Testing. Additionally, EC7 is supplemented by national annexes allowing each member country to stipulate their safety factors in line with national safety standards or recommend alternative design procedures. One of the primary goals of the Eurocodes, including EC7, is to standardize and harmonize geotechnical design across Europe. This is achieved through the consistent implementation of limit state design, aligning diverse national practices into a unified framework.

Eurocodes allow to achieve the target reliability level by Using Partial Safety Factors method: a semi-probabilistic approach for design, which involves the application of partial safety factors, the most widely used method in practice. It is a very practical approach that applies factors to account for uncertainties in loads, material properties, and construction practices. They are used to increase the nominal values of loads and to decrease the nominal values of material strengths. The Eurocodes require consideration of various load combinations (permanent, variable, and accidental) and apply different partial safety factors for these, reflecting their different levels of uncertainty and impact on overall reliability. The values of the partial safety factors in the Eurocodes are calibrated based on achieving a target reliability level, which is typically expressed in terms of a probability of failure over the structure’s lifetime. the safety factors are deterministic. EC7 only provides a framework in which each member state defines the design approach as well as the partial factors according to national design and safety requirements. The design approaches differ in the way partial factors are applied and lead to remarkably different designs especially in the case of shallow foundations under complex loading. Hence, the authors believe that the actual safety of the foundation cannot be reliably determined with EC7.

The Eurocodes, however, allow engineers to use fully probabilistic design, too. While the partial safety factors method provides a straightforward and standardized approach to ensure safety, the full probabilistic design offers a more accurate, flexible, and potentially cost-effective alternative, particularly suitable for complex or unique engineering challenges. Another important application domain are projects where a specific design is replicated numerous times, the adoption of a full probabilistic design approach offers substantial long-term benefits. This is particularly relevant in achieving cost-effectiveness and efficiency over the lifecycle of these structures. The initial investment in a more refined, full probabilistic design can be amortized over all these instances. The increased upfront effort and cost associated with this detailed analysis become relatively minor when distributed across multiple applications. Full probabilistic design allows for more precise optimization of materials. Even marginal savings per unit, when multiplied by the number of repetitive structures, can lead to significant overall cost reductions. This optimization not only lowers material costs but also can reduce transportation and labor expenses. Moreover, while each site may have unique environmental and loading conditions, a probabilistic approach can tailor the design to these specifics, enhancing safety and reliability. This adaptability ensures that each instance of the replicated structure is optimized for its unique conditions, rather than relying on a one-size-fits-all approach. A probabilistic approach provides a more accurate assessment of risks and uncertainties, which is crucial in repetitive structures where the impact of any design flaw is multiplied. This can reduce the risk of failure and associated liabilities, offering a more sustainable and responsible engineering solution. Finally, the accessibility of advanced computational tools and software (see e.g.^14^) has made full probabilistic design more feasible and cost-effective, even for large-scale projects involving repetitive structures.

The full probabilistic design approach can be found already in Eurocode EN 1990: Basis of structural design^15^. In the Czech Republic, an older code that allowed for a fully probabilistic design already existed: ČSN 73 0031(1988)^16^. This document, however, was not used very often and the full probabilistic design did not become a standard. Not only the Czech engineering community has started start to realize this fact, and now we can see more projects dealing with this topic (see e.g.^17–25^). The importance of fully probabilistic calculations lies in the rational and mathematically sound consideration of randomness and uncertainty due to a random and uncertain character of inputs to the model of a structure.

The ongoing discourse on the comparison between partial safety factors and full probabilistic design, particularly in the context of structural reliability analysis, is not restricted to geotechnical structures in Europe; see, e.g., a study^26^ related to reinforced concrete frame structures in China. These studies offer valuable perspectives on how design values for actions and resistance are calculated and the implications of these methodologies on the overall safety and efficiency of structures.

The Czech standard ČSN 731001 implemented the partial safety factor method before many other European countries adopted similar approaches in their national standards or transitioned to the Eurocodes. The ČSN 731001, particularly in its earlier versions, was ahead of its time in integrating the partial safety factor method into structural design, including foundation design. The main topic of the present paper is the comparison of the shallow foundation design according to EC7 and ČSN 73 1001 with a rigorous check of the foundation design reliability using fully probabilistic calculation. The paper proceeds with a detailed presentation of a demonstrative case study on a shallow foundation. Initially, it offers a succinct overview of the partial safety factor method. This is followed by an in-depth definition of the probabilistic model and the performance function specific to the foundation. The methods utilized for analyzing the failure probability, as well as the sensitivity of this probability to the input variables, are also thoroughly explained. In the discussion section, the paper highlights the critical significance of distribution tails, particularly in the realm of refined analysis. This section underscores how understanding the tail behavior of distributions can impact the accuracy and reliability of probabilistic assessments in geotechnical engineering.

### Shallow foundation design according to Eurocode EC 7 and ČSN 73 1001

**Figure 1:**
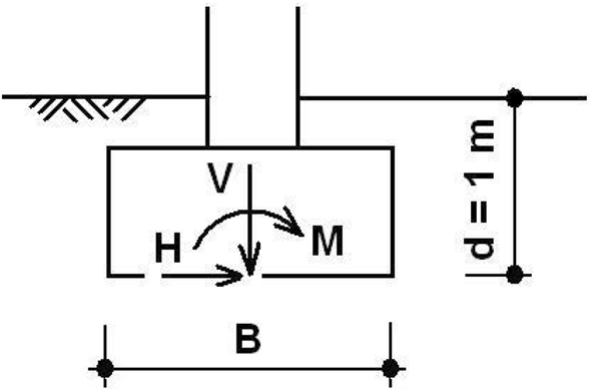
Shallow foundation—schema.

In this section, a comparison of shallow foundation design according to EC7^13^ and ČSN 73 1001^27^ is presented. EC 7, incontrast to ČSN 73 1001, defines three design approaches (DA) which are different in defining of the partial factors of loads, materials and resistance. The Limit state method was used for design shallow foundation before acceptance of EC7 in the Czech Republic. From all three DA the authors decided for using the DA3, which is very similar to the Czech standard. This decision ensued also from own experiences and the experience gained from other comparative studies^28,29^. The design of a shallow foundation will now be illustrated using the model example adopted from a previous study performed in^30^. In our problem the strip footing with depth of 1 m below the level of the final grade is considered. The resultant of loads in the base of footing is defined by three components: vertical force (*V*), horizontal force (*H*) acting in the direction of the strip footing width (*B*) and bending moment (*M*) that is also acting in the direction of the strip footing width, see Fig. 1. The given characteristic value of the vertical force is 400 kN/m. The horizontal force is variable limited by a maximum value of the horizontal/vertical force ratio of 0.577. This limit value represents an inclination 30° of resultant from the vertical direction. The magnitude of the bending moment is considered to be the quadruple of the horizontal force:

1 $$M_{k} = 4H_{k} \,and\, M_{d} = 4H_{d} ,$$

where *M*_k_ (*M*_d_) is the characteristic (design) value of the bending moments and *H*_*k*_ (*H*_*d*_) is the characteristic (design) value of the horizontal force. The design value of the effect of action (*E*_*d*_) is represented by the contact stress at the foundation level.

2 $$E_{d} = V_{d} /\left( {B - 2e} \right) = V_{d} /b_{ef} ,$$

where *V*_*d*_ is the design value of the vertical force, *B* is the foundation width and *e* is the resultant eccentricity of the load at the footing bottom. The design value of the vertical force is taken as γ_*F*_ = 1,35 (or γ_*F*_ = 1,00) multiple of its characteristic value (note: the partial factor γ_*F*_ = 1,00 will not be considered in the probabilistic analysis from here on). It results from recommendation^30^. The Czech standard (ČSN) considers the partial factor of γ_F_ = 1,20. The foundation soil is represented by coarse-grained soil (sand) with the following parameters: the characteristic value of effective angle of internal friction *ϕ*_k_ = 32.5° and the unit weight γ = 19 kN/m^3^^31^. In EC7, the design value of the friction angle is calculated with using the following formula

3 $$\phi_{d} = \arctan \left[ {\tan \left( {\phi_{k} } \right)/1.25} \right].$$

In ČSN 73 1001, the design value of angle of internal friction is not calculated from the angle tangent but directly from the angle. From this reason the design value is *ϕ*_d_ = 32.35° (according to ČSN 73 1001) as opposed to *ϕ*_d_ = 27° in EC7. The design value *ϕ*_d_ was not determined from *ϕ*_k_ = 32.5° but from the mean value of 36.35°. The difference is in determination of characteristic values by ČSN (mean value) and EC7 (5% percentile) for shallow foundation. The design resistance (bearing capacity of soil) is calculated with using the following formula, which is defined in ČSN 73 1001 (also known as Brinch Hansen, 1968–1970, equation)

4 $$R_{d} = c_{d} .N_{c} .s_{c} .d_{c} .i_{c} + \gamma_{1} .d.N_{d} .s_{d} .d_{d} .i_{d} + \gamma_{2} .\frac{{b_{{{\text{eff}}}} }}{2}.N_{b} .s_{b} .d_{b} .i_{b} .$$

In view of the fact that this formula is slightly different in ČSN 73 1001 and EC7 D annex, formulas used during calculation will be specified. In Eq. (4), the symbol *c*_*d*_ has the meaning of the design value of the effective cohesion, γ_*1*_ a γ_*2*_ are the unit weights above and beneath the foundation bottom (in our case γ_*1*_ =  γ_*2*_ = 19 kN/m^3^) and *b*_*ef*_ is the effective foundation width. Bearing capacity factors (*N*_*d*_*, N*_*b*_), shape factors (*s*_*d*_*, s*_*b*_), depth factors (*d*_*d,*_* d*_*b*_) and inclination of load factors (*i*_*d*_*, i*_*b*_) are in the non-zero summands calculated according to these formulas

5a $$N_{d} = \tan^{2} \left( {45^\circ + \frac{{\phi_{d} }}{2}} \right) \times \exp \left( {\pi \tan \phi_{d} } \right),\quad N_{b} = 1.5\left( {N_{d} - 1} \right) \cdot \tan \phi_{d} ,$$

5b $$s_{d} = s_{b} = 1,$$

5c $$d_{d} = 1 + 0.1\sqrt {\left( {\frac{d}{{b_{ef} }}} \right)\sin \left( {2\varphi_{d} } \right)} , d_{b} = 1,$$

5b $$i_{d} = i_{b} = \left( {1 - H_{d} /V_{d} } \right)^{2}$$

Considering all these assumptions and limiting conditions, the width of shallow foundation width *B* was calculated for a range of values of the horizontal force. We consider the most economical design, where the equality between the bearing capacity design value and the loads effect design value is accurately carried out, i.e.

6 $$E_{d} = R_{d} .$$

**Figure 2:**
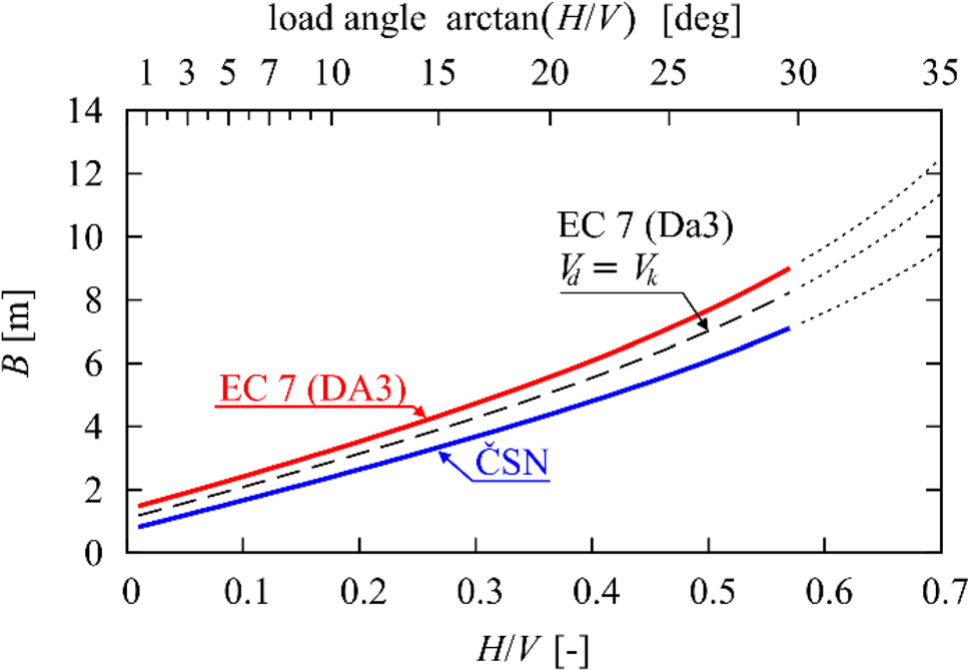
Shallow foundation—widths designed with EC7 (DA3) and ČSN 73 1001.

The results of these designs are summarised in Fig. 2, where the foundation width is the function of only the ratio between the horizontal and vertical forces.

For the numerical purposes of verification using the fully probabilistic approach, the designed width of the foundation under consideration, based on both EC7 (DA3) and the older Czech code ČSN, has been approximated, for the studied wide range of ratios between the horizontal and vertical force, using the linear regression on a polynomial function of the 4^th^ order as

7 $$B \approx \mathop \sum \limits_{i = 0}^{4} a_{i} \left( {\frac{{H_{d} }}{{V_{d} }}} \right)^{i} ,$$

where the vector of regression coefficients **a** for the design according to EC and ČSN read:

8a $${\varvec{a}}_{EC} = \left\{ {1.4126;8.8832;12.9157; - 28.8695;34.9449} \right\},$$

8b $${\varvec{a}}_{{\check{C}SN}} = \left\{ {0.7474;8.4467;7.8982; - 17.7385;21.4826} \right\}.$$

The numerical accuracy of the linear regression has been verified using the coefficient of determination that exceeds 0.99999 in both cases (in the range of *H/V* ∈(0;0.7)). The validity of the approximation has also been checked using GEO 5 computer code^32^. Note that the absolute term of the EC approximation ($a_{0} = 1.4126$) is about twice as big as the corresponding term for the ČSN approximation (0.747). This means that for very small load angles the EC yields about twice wider foundation compared to ČSN, see Fig. 2.

### Reliability verification of the design using the fully probabilistic approach

This section presents the verification of failure probability of the shallow foundation by using the fully probabilistic approach. The designs according the EC7 and ČSN are represented by Eq. (6) which is approximated by Eqs. (7) and (8). Before showing the results of the verification, the problem must redefined in a probabilistic model. This is what follows.

### Definition of the probabilistic model of the foundation

The analysis considers the following vector of three basic random variables

9 $${\varvec{X}} = \left\{ {\phi ,V,H} \right\},$$

collecting the angle of friction of the soil *ϕ*, random vertical *V* and horizontal *H* components of the load in the footing bottom. The resulting random moment is, in accordance with the previous analysis (Eq. (1)), considered as a function of the horizontal force: *M* = *4H* and so it becomes random, too. This dependency also determines another derived random variable: the eccentricity of the resultant in the footing bottom.

The safety margin (random variable *Z*) is defined as a function of the three random variables:

10 $$Z = g\left( {\varvec{X}} \right) = g\left( {\phi ,V,H} \right).$$

The task is to determine the probability of exceeding the load-bearing capacity of the soil, i.e., the probability of *Z* = *g*(**X**) being negative.

11 $$p_{f} = P\left( {g\left( {\varvec{X}} \right) \le 0} \right) = \iiint\limits_{{X \in D_{f} }} {f\left( {\varvec{X}} \right)\;d\phi \;dV\;dH},$$

where *D*_*f*_ denote the region in which *g*(**X**) ≤ 0 (failure region) and *f* (**X**) is the joint distribution function (PDF) of random vector **X**. The safety margin *Z* is defined as the difference between a random model resistance of the soil *R* and random model load effect *E*. The resistance *R* is calculated analogously to the partial safety factor approach presented in the previous section (Eq. (4)) with one basic difference: instead of using the design values obtained by application of the safety factor the resistance is evaluated directly using the random variables. Therefore, the resistance is defined as

12a $$R = \gamma \left( {1 - \frac{H}{V}} \right)^{2} \left\{ {d \times N_{d} \times \left[ {1 + 0.1\sqrt {\frac{d}{{b_{ef} }}\sin \left( {2\phi } \right)} } \right] + \frac{{b_{ef} }}{2}\left[ {1.5\left( {N_{d} - 1} \right)\tan \phi } \right]} \right\},$$

12b $$N_{d} = \tan^{2} \left( {45^\circ + \frac{\phi }{2}} \right){\text{exp}}\left( {\pi \tan \varphi } \right).$$

For any acceptable ratio of *H/V*, the width of the foundation is obtained using the aforementioned approximation of the design according to the standards (Eq. (7)).

The load effect is modelled as (compare with Eq. (2))

13 $$E = V/\left( {B - 2H/V} \right) = V/b_{ef} .$$

An important part of the probabilistic model is the joint probability density function (PDF) of the basic random variables. In an ideal situation, the PDF is obtained directly from the in-situ measured data. For the purpose of the present study, the authors judged about the PDF based on their experience. The reason for doing so is that no real situation is studied. Rather, a model analysis of a particular type of foundation is examined.

**Figure 3:**
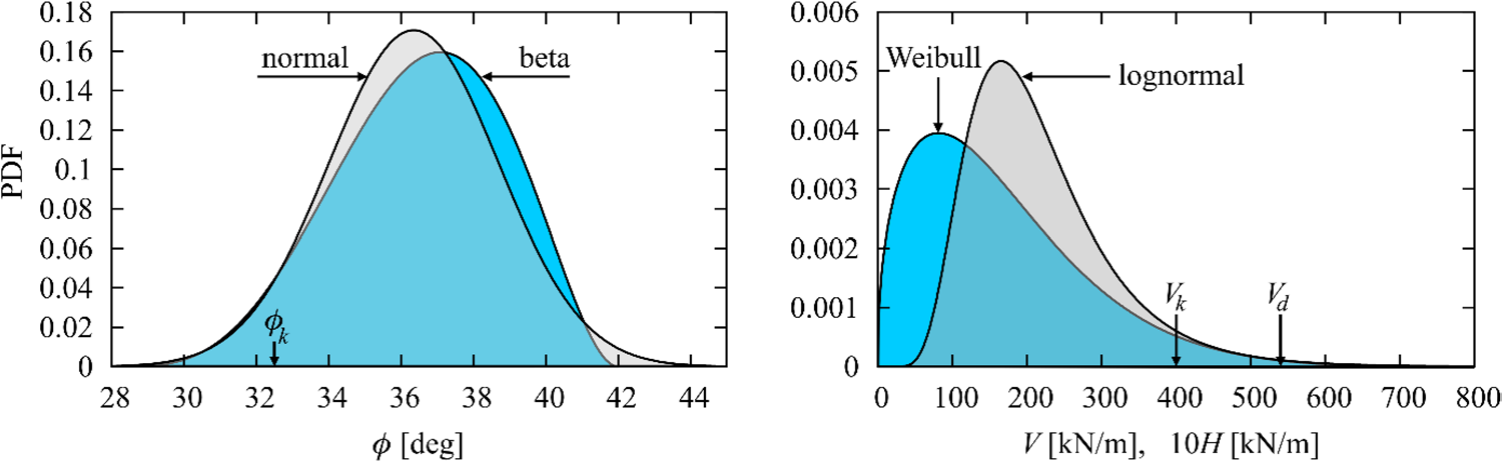
Probability density functions (PDF) of the basic random variables. Left: normal and beta distributions of the fiction angle. Right: lognormal and Weibull distributions of the horizontal and vertical forces. The common characteristic and design values are highlighted.

Two alternatives of the joint probability density of the three random input variables are studied. The first one features normal and lognormal variables only as summarized in the first three rows of Table 1. The angle of internal friction is modelled as a normal random variable with parameters enumerated in the first row of Table 1. These two adjustable parameters (degrees of freedom defining the distribution) were set to fulfil two requirements: the characteristic value used above (32.5°) remains the 5% percentile and the coefficient of variation (cov) is kept at 6.43% which corresponds to the experience of the authors and is in accordance with the data in literature. Both the horizontal and vertical forces are assumed lognormally distributed (see rows 2 and 3) with the characteristic value (95% percentile) equal to 400 kN/m and design value (99% percentile) equal to 540 kN/m. The lognormal shape has been selected for two reasons: it is a common choice and it enables only positive values of the loads. The ratio between the mean values of *V* and *H* is 10 and the shapes of the distributions are identical, see Fig. 3 right.

**Table 1:** Definition of basic random variables. Forces *V* and *H* can only be positive.

No	Name	Notation	Unit	PDF shape	Mean value	Standard deviation	cov [%]	Skewness	Kurtosis excess
1	Angle of friction	*ϕ*	deg	normal	36.350	2.337	6.430	0	0
2	Vertical force	*V*	kN/m	lognormal	217.000	97.000	44.700	1.430	3.845
3	Horizintal force	*H*	kN/m		21.700	9.700	44.700		
4	Angle of friction	*ϕ*	deg	Beta	36.545	2.315	6.330	− 0.275	− 0.458
5	Vertical force	*V*	kN/m	Weibull	169.000	119.290	70.590	1.1479 (*m* = 1.438)	1.653
6	Horizintal force	*H*	kN/m		16.900	11.929	70.590

In the second alternative, the angle of friction was modelled as beta distributed (a distribution model with a limited support from 28 to 42 degrees), and the horizontal and vertical forces were modelled using the two-parameter Weibull distribution, which allows occurrence of positive values only.

In order to achieve meaningful correlations between the horizontal and vertical components of the load forces, a strong positive statistical correlation between them was assumed. The two forces *V* and *H* have correlation coefficient of 0.8. Such a strong correlation limit the number of occurrences of a low vertical force being combined with a high horizontal force: a combination that would have to be assessed using a shear criterion, not normal stress in the footing bottom. The most frequent ratio approximately equals the ratio of the mean values (0.1) which corresponds to the resultant angle of 5.7°. This mimics the most frequent situation in practice. Note that the strong statistical correlation still permits occurrence of other ratios. However, samples with ratios exceeding 0.577 (load angle of 30°) are automatically censored and the corresponding results are disregarded.

The friction angle is modelled as independent of V and *H*. The joint probability density is modelled using the Nataf model^33,34^ and simultaneously numerically using the optimization algorithm^35^.

The probabilistic model has been defined and solved using FREET software^36^.

### Solution methods

The probability of failure of the foundation designed using the two design standards has been calculated by using both, direct simulation based on Monte Carlo type sampling and by approximation using FORM method^37^.

The *simulation-based approach* estimates the failure probability (Eq. (11)) by sampling the triplets of **X** =  {*ϕ*, V, H} from the joint PDF, evaluating *g*(**X**) and counting the number of negative results *N*_*f*_ (failures). The probability estimate reads

14 $$p_{f} \approx \frac{{N_{f} }}{{N_{{{\text{sim}}}} }},$$

where *N*_sim_ is the total number of samples. In practice, this estimate requires a large number of simulations to yield statistically significant results. The reason is that the target failure probabilities are very low. Sampling from the vector ${\varvec{X}}$ can be performed by crude Monte Carlo or e.g. by Latin Hypercube Sampling (LHS). Both methods were used in the present study. We remark that a particularly effective method for the estimation of failure probability is to use active learning strategy for the selection of points for the evaluation of the full model, and then perform importance sampling analysis of a surrogate model build with the help of the optimal training points^38^. In the present case, the evaluation of the full model is so quick, that these advanced techniques are not necessary.

The *FORM approximation* estimates the failure probability by linearization of the failure surface *g*(**X**) = 0 in the vicinity of the design point (most probable point of failure lying on the limit state curve). Once this linearization is performed in the transformed **U**-space of random variables, the failure probability can be estimated very easily (see e.g.^37^). The transformed space **U**-is a space of uncorrelated standardized space of normal variables. The isoprobabilistic transformation between the physical space of **X** and the **U**-space is performed via the Nataf transformation here. Due to the rotational symmetry of the joint normal PDF in the **U**-space, the failure probability is estimated as

15 $$p_{f} \approx \Phi \left( { - \beta } \right),$$

where Ф (∙)is the normal cumulative density function, and *β* is the so-called *Hasofer-Lind* (HL) *reliability index*. Geometrically, *β* is the smallest distance from the origin in **U**-space to the design point (a point on lying on *g*(**X**) = 0).

Let us call denote x^*^ the design point in the physical space and u^*^ in the **U**-space. Then HL-index *β* is calculated as

16 $$\beta = \sqrt {\left( {{\varvec{u}}^{*} } \right)^{T} {\varvec{u}}^{*} } .$$

Coordinates of the design point in the **U**-space can be written as a function of index *β* and a vector of sensitivities***α***:

17 $${\varvec{u}}^{*} = \beta {\varvec{\alpha}}\quad \Leftrightarrow \quad \beta = {\varvec{\alpha}}\left( {{\varvec{u}}^{*} } \right)^{T} = \beta \user2{\alpha \alpha }^{T}$$

The second equality holds because the sensitivities have unit Euclidean size: $\sqrt {\user2{\alpha \alpha }^{T} } = 1$. The geometrical meaning of the sensitivities in the **U**-space is that they show the direction of the design point with respect to the origin. If this unit-size vector (normal to the failure surface) is multiplied by the length *β*, one obtains the design point, see Eq. (17). These sensitivities are obtained using the gradient vector

18 $$\nabla g\left( {\varvec{u}} \right) = \left( {\frac{\partial g}{{\partial u_{1} }}\left( {\varvec{u}} \right),\frac{\partial g}{{\partial u_{2} }}\left( {\varvec{u}} \right),\frac{\partial g}{{\partial u_{3} }}\left( {\varvec{u}} \right)} \right),$$

19 $$as\, \user2 { \alpha } = - \frac{{\nabla g\left( {\varvec{u}} \right)}}{{\left| {\nabla g\left( {\varvec{u}} \right)} \right|}}.$$

From the definition of $\beta$ as the length of the vector ${\varvec{u}}^{*}$ it follows that

20 $$\frac{{\partial \beta \left( {{\varvec{u}}^{*} } \right)}}{{\partial u_{i} }} = \frac{\partial }{{\partial u_{i} }}\left( {\mathop \sum \limits_{j = 1}^{3} u_{j}^{2} } \right)^{1/2} = \alpha_{i}$$

From the above, the numerical value of α_i_ is thus a measure of the sensitivity of the reliability index to inaccuracies in the value of *u*_*i*_ at the design point u^*^ (direction cosines).

## Results and discussion

In the studied case, the failure surface is very simple in the physical space and it is almost linear. There is just a single design point and thus the FORM approximation is very accurate. As will be shown later, the probabilities of failure obtained with FORM and sampling analysis are comparable and this leads to a conjecture that the failure surface is not very curved in the **U**-space.

**Figure 4:**
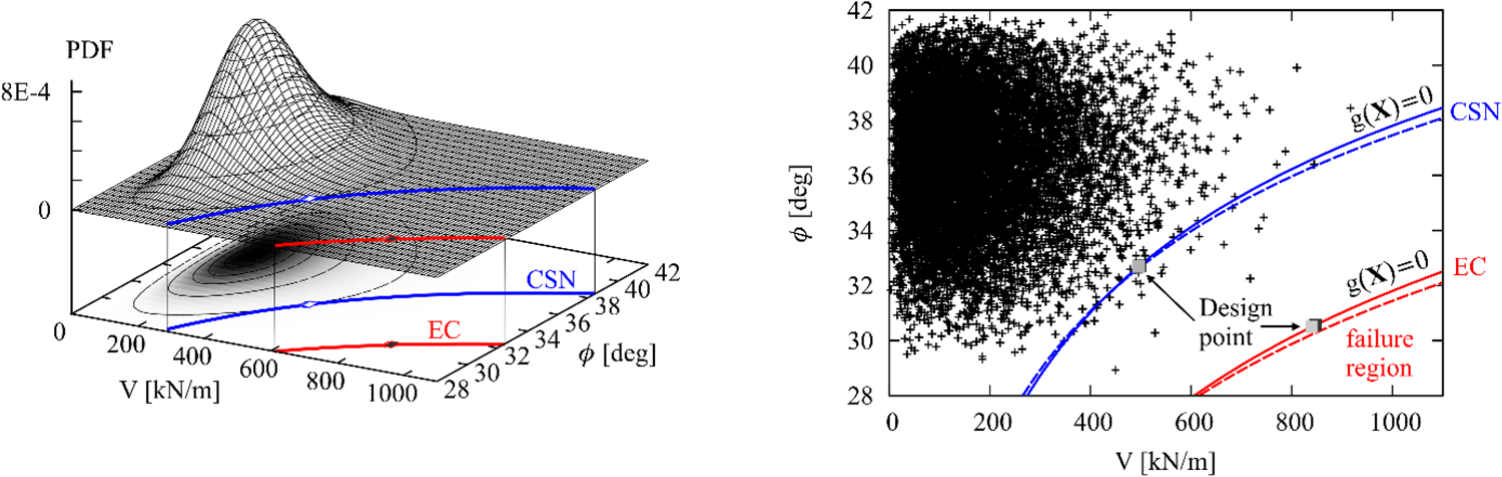
The joint PDF of the random vector and the failure surfaces g(X) = 0 with the design point. The solid line of the failure surface corresponds to H/V = 0.1, the dashed line corresponds to H/V = 0.5. Left: visualization of the joint PDF for the input alternative Normal-Lognormal-Lognormal (the first three rows of Table 1). Right: positions of 10,000 sampling points from the alternative input Beta-Weibull-Weibull PDF.

Figure 4 visualizes the situation for a fixed value of horizontal force *H*. The left part of the figure shows the two-dimensional joint PDF of independent angle of the friction ф and the vertical force *V*.

The following Table 2 summarizes results of the design point search. The first row shows the coordinates for the case of width B designed according to the EC7 (DA3). From the numerical values of sensitivities (the last three columns) it can be seen that the safety index *β* is not very sensitive to variations in the horizontal force *H*. Most of the sensitivity is on the vertical force *V* and friction angle *ϕ*. These sensitivities are summarized in the rightmost part of Table 2. It can be seen that neither the choice of the design code nor the alternative of the joint probability density of the input vector has a significant impact on the sensitivity.

**Table 2:** Results of FORM analysis—design point.

*B* design with	PDF alternative	Design point x*				Design point u*			Sensitivities α		
		*x*_1_ = *ϕ*	*x*_2_ = *V*	*x*_3_ = *H*	*H*/*V*	*u*_1_ (*ϕ*)	*u*_2_ (*V*)	*u*_3_ (*H*)	*α*_1_ (*ϕ*)	*α*_2_ (*V*)	*α*_3_ (*H*)
EC	N,Lg,Lg	30.57	846.92	64.76	0.08	− 2.474	3.404	0.007	− 0.588	0.809	0.002
EC	B,We,We	30.50	839.71	67.34	0.08	− 2.630	3.595	0.004	− 0.590	0.807	0.001
**ČSN**	**N,Lg,Lg**	**32.72**	**498.09**	**42.02**	**0.08**	**− 1.553**	**2.160**	**0.005**	**− 0.584**	**0.812**	**0.002**
**ČSN**	**B,We,We**	**32.69**	**496.00**	**41.59**	**0.08**	**− 1.564**	**2.128**	**0.006**	**− 0.592**	**0.806**	**0.002**

Let us now present the values of the safety index *β* and the corresponding failure probabilities. The left part of Table 3 presents these values obtained by FORM linearization in the design points given in Table 2. For comparison purposes, the tables on the right-hand side display results from the sampling analysis conducted with 100 million Monte Carlo (MC) samples and 15 million LHS samples.

**Table 3:** Failure probabilities and safety indices obtained by FORM and sampling analysis.

*B* design with	PDF alternative	FORM analysis		MC analysis		LHS analysis	
		*β*	*p*_f_ = Φ(-*β*)	*p*_f_ = *N*_f_ /*N*_sim_	*β*	*p*_f_ = *N*_f_ /*N*_sim_	*β*
EC	N,Lg,Lg	4.208	1.29E-05	1.04E-05	4.256	1.08E-05	4.248
EC	B,We,We	4.455	4.20E-06	1.80E-06	4.633	2.00E-06	4.611
**ČSN**	**N,Lg,Lg**	**2.660**	**3.91E-03**	**3.55E-03**	**2.692**	**3.79E-03**	**2.671**
**ČSN**	**B,We,We**	**2.641**	**4.14E-03**	**3.05E-03**	**2.743**	**3.31E-03**	**2.715**

It can be seen that, in basic alternative of design via EC7 (DA3) and input set designated as N-Lg-Lg, the failure probability of the shallow foundation is about *p*_f_ = 1.3 × 10^−5^. This corresponds to the safety index *β* of ca 4.2. Such a failure probability about equal to the target value for this kind of structures. The target reliability indices are found by the analysis of observed failure rates and the outcomes of the cost–benefit analyses^39^. The central value (*β* = 4.2) should be considered as the most common design situation. This result is less conservative than the usual safety targets, but the difference is not great. For example, in the Eurocode the value of *β* = 3.8 (*p*_f_ ≈ 0.7 × 10^−5^) is mentioned for a reference period of 50 years. If the failure events in the various years are assumed to be mutually independent, this correspond to a nominal yearly failure rate of 1.3 × 10^−6^ (*β* = 4.7). As complete independency of resistance and loads in subsequent years is not realistic, the Eurocode target could better be interpreted as corresponding to *β* = 4.5 for 1 year.

One can conclude that a design based on EC7 (DA3) yields a well-balanced design between safety requirements and economical demands. It must be taken into the account, though, that engineers tend to slightly overdesign the foundation width *B* from various reasons (either rounding to integer dimensions or for psychological reasons). In practice, therefore, the designed widths might be somewhat greater than the perfect design studied here, in which *E*_*d*_ = *R*_*d*_ (see Eq. (6)). In consequence, the failure probabilities are even lower in real situations.

It is interesting to compare the reliability of shallow foundation designed using EC7 (DA3) and the Czech standard ČSN. It was shown above that the lowest necessary width *B* required by ČSN is considerably smaller than if EC7 is used (in the studied case). This reduction in width must have an effect on the foundation reliability when the same random inputs are used. Indeed, the probability of failure of foundation is about three orders of magnitudes less! This can be seen in Table 3 (rows with bold background). The radical decrease of safety holds for both studied alternatives of the input PDFs. The fact that the failure probability is much greater can also be deduced from the comparison of design points in Table 2. While the coordinates of ф and *H* are almost identical for both designs, the coordinates of *V* is considerably different. The situation is clearly illustrated in Fig. 4 in which the failure surface for ČSN-designed foundation is much closer to the average inputs than the one for EC7 design.

One can doubt about the effect of the assumed strong correlation between the horizontal and vertical load components. We have performed numerical analyses with various correlations ranging from 0 to 1 and the results (failure probabilities and design point locations) remain almost unaffected.

In order to see whether there is any impact of the assumed distributions of **X** = {*ϕ*, V, H}, we have performed a simultaneous analysis with another set of input distributions. These are summarized in Table 1, rows 4 to 6. The shear angle was considered *beta* distributed within the range of 28°—42° (the shape parameters are: 4.6988 and 3, see Fig. 3 left). The characteristic value of 32.5 corresponding to probability of 5% was preserved. The vertical and horizontal forces are Weibull distributed with the shape parameter *m* = 1.438, see Table 1 and Fig. 3. Again, the parameters of the distributions of forces were set to keep the same characteristic and design values (400 and 540 kN/m) as in the preceding analyses. This makes all the alternatives under comparison compatible. While there was almost no difference in the position of the design point (Table 3, second row), the failure probabilities changed almost an order of magnitude. The reason is that the Weibull and lognormal distributions differ in shape appreciably. Weibull distributions have much smaller modes than the lognormals and therefore the most frequent values of the forces are lower. On the other hand, lognormal distribution has slightly heavier upper tail and there is a greater probability of occurrence of a large force. Also the beta distribution of the angle of friction tends to yield greater values more frequently while the angle never drop below 28° (see the greater modus in Fig. 3 left). All these aspects are the reason why the alternative with Beta-Weibull-Weibull PDF yields much lower failure probabilities, see Table 3.

It should be noted that the sensitivities ${\varvec{\alpha}}$ obtained above by finding the design point in FORM represent sensitivities of the *β* index on random inputs. Another sensitivity measure can be obtained by calculating the statistical correlations between inputs and *g*(**X**) = 0 in the sampling analysis. These correlations quantify sensitivities of the output on variations in inputs, mostly in the vicinity of the mean values. Table 4 present the correlations and it can be seen that both studied types of design (EC and ČSN) and also both types of input distributions yield the same results: it is mainly the angle of friction that influences the output because the correlations are close to 1. Other parameters have the absolute values of correlations of ca 0.3 or less and therefore their influence can be considered as low.

**Table 4:** Sensitivities (correlation coefficients between random inputs and *g*(**X**)) for foundation designed with EC and ČSN and for both alternatives of PDF of the input random vector.

*B* design with	N,Lg,Lg			B,We,We		
	*x*_1_ = *ϕ*	*x*_2_ = *V*	*x*_3_ = *H*	*x*_1_ = *ϕ*	*x*_2_ = *V*	*x*_3_ = *H*
EC	0.99	− 0.12	− 0.14	0.95	− 0.11	− 0.17
**ČSN**	**0.94**	**− 0.29**	**− 0.29**	**0.89**	**− 0.30**	**− 0.34**

**Figure 5:**
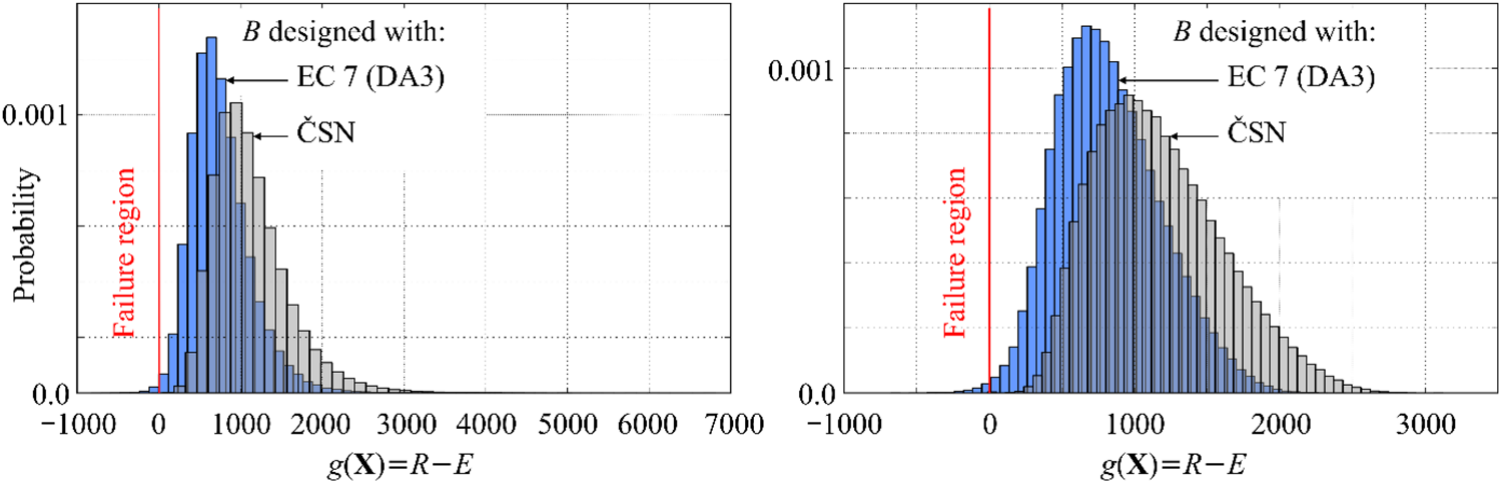
Empirical histograms of the safety margin. Left: Input parameters Normal-Lognormal-Lognormal. Right: Input alternative Beta-Weibull-Weibull.

Finally, Fig. 5 presents the empirical histograms of the safety margin—random variable *Z.* It can be seen that for all studied combinations of inputs PDFs and design alternatives (EC7 and ČSN), the distribution of safety margin is skewed and thus far from normally distributed. This explains why the Rjanitzyne-Cornell safety index calculated from the mean and standard deviation of $Z$

21 $$\beta_{RC} = \frac{{\mu_{Z} }}{{\sigma_{Z} }} = \frac{1}{{cov_{z} }}$$

is inappropriate in this case.

## Conclusions

The changes in the design codes for foundation structures in Europe over the past two decades have been pivotal in enhancing the safety, sustainability, and innovation in structural engineering practices. They reflect a continuous effort to incorporate the latest scientific knowledge and technology, as well as to respond to the evolving needs of society and the environment. While the Eurocodes integrate probabilistic concepts, particularly in the form of partial safety factors, they primarily represent a semi-probabilistic approach, with options for more detailed probabilistic analysis where needed. In this context, one of the change is the inclusion of advanced analytical and numerical methods: the codes now recognize and include more advanced methods for design and analysis, acknowledging the advancements in computational capabilities. This change allows engineers to use more precise and sophisticated methods in their design work. The development of advanced statistical methods applied in the field of structural reliability open the door for the full probabilistic design which combine the advanced definition of probabilistic model with the refined computational models to achieve the desired material saving, better control over the final reliability level and detailed insights into the probabilities of different failure modes, enabling engineers and decision-makers to make better-informed choices about risk management and safety measures. Today, full probabilistic analysis transcends theoretical exercise, becoming a practical and realizable tool in the engineering domain.

In this paper, the design of a shallow foundation is analyzed in accordance with the ultimate limit state as prescribed by Eurocode 7 (EC7). The initial step involves determining the most economical design permissible under both EC7 and the older Czech standard (ČSN). Following this, the second step entails a verification of the reliability of this design through a comprehensive fully probabilistic simulation.

The following conclusions are formulated from this study:

- The design of the width of shallow foundation by EC7 (DA3) has lead to the same value as if the full probabilistic method was used. The conclusion is made by checking the probability of failure of the foundation.
- The calculated probabilities of failure of foundation designed by EC7 or ČSN represent the lower bound on the failure probabilities because the width of the foundation can only be exceeded. The design considered here was always the most economical design allowed by the code.
- The results carried out by using simulation approaches (MC, LHS) or FORM method show that probability of failure is not too much affected by the statistical correlation between the vertical and horizontal components of the load. From this point of view the design by EC 7 yields a well-balanced level of reliability for a wide range of ratios between horizontal and vertical forces.

To have an idea about the influence of density selection on the input, the loading distribution and the soil friction angle distribution was changed. This change has lead to failure probability of a foundation designed with EC7 about one order of magnitude lower and thus the width would have to be increased to achieve the target reliability. This observation was, however, only possible with the full probabilistic computation. In the partial safety factor method, the designers have no means to alter the (fixed) partial safety factors based on detailed information on the parameter distribution, especially in the tails. Therefore, in cases when refined input data are available, the full probabilistic design offers more nuanced design with the potential of more cost-effective safe design.

## Data Availability

The datasets generated during and/or analyzed during the current study are available from the corresponding author on reasonable request.
